# Types of Nutrition Knowledge, Their Socio-Demographic Determinants and Their Association With Food Consumption: Results of the NEMONIT Study

**DOI:** 10.3389/fnut.2021.630014

**Published:** 2021-02-12

**Authors:** Franziska Koch, Ingrid Hoffmann, Erika Claupein

**Affiliations:** Institute for Nutritional Behaviour, Max Rubner Institute, Karlsruhe, Germany

**Keywords:** nutrition knowledge, types of knowledge, food consumption, dietary recommendations, healthy eating, NEMONIT

## Abstract

**Objectives:** To investigate nutrition knowledge in the German population, its determinants and its association with food consumption.

**Methods:** Data were obtained from the NEMONIT study (2014/15, *n* = 1,505, participants' age: 22–80 years). Nutrition knowledge was measured using the consumer nutrition knowledge scale (CoNKS) in a computer-assisted telephone interview. Two 24-h recalls were conducted to assess food consumption, which was evaluated using the Healthy Eating Index-NVS II.

**Results:** Areas for knowledge enhancement were the understanding of health benefits of fruit and vegetable consumption, the concept of a balanced diet and saturated fatty acids. Nutrition knowledge was higher among females, younger and high socio-economic status participants. Correlations between nutrition knowledge and a favorable diet were significant but low. Analyses of types of nutrition knowledge yielded similar results for procedural knowledge and knowledge on nutrients but not for knowledge on calories.

**Conclusions:** Areas for knowledge enhancement were identified, but an increase in nutrition knowledge alone seems unlikely to result in large improvements of dietary behavior.

## Introduction

The factors influencing an individual's food choice are numerous and include, amongst others, habits, practical skills, cultural or environmental factors as well as motives like taste, convenience or price (1–3). Nutrition knowledge might also be one of several factors influencing food choice. Given the abundance of possibilities for food choices in Western societies, a certain level of knowledge might be necessary to make a healthy selection. Since food consumption of Western populations is often not in line with dietary recommendations (4–7), knowledge dissemination and nutrition education are common–but usually not evaluated–strategies of public health initiatives aiming to change dietary behavior.

As reported in a systematic review by Spronk et al. (3), it is well documented in international studies that nutrition knowledge is influenced by age, sex, and socio-economic status. However, the direction of the relationship between age and nutrition knowledge was contradictory across studies. While Hendrie et al. (8) observed a positive relationship, Dickson-Spillmann and Siegrist (9) and Dickson-Spillmann et al. (2) observed a negative relationship and Parmenter et al. (10) reported a curvilinear relationship, where middle-aged groups had a higher knowledge than younger and older participants. It should be noted though that the studies differ substantially in how they defined the age groups, thus limiting comparability. With regard to the association of nutrition knowledge and food consumption the results are also inconclusive. Although the review by Spronk et al. (3) shows that the majority of studies found significant associations between higher nutrition knowledge and healthier food consumption, especially higher fruit and vegetable consumption, these associations were rather weak.

A limitation of previous studies is the challenging measurement of nutrition knowledge. The General Nutrition Knowledge Questionnaire (GNKQ) has been well studied, validated, and adapted to different populations (11–15), but is far too long to be used as one of many instruments in a population-wide survey. Although many recent studies address the development and validation of questionnaires for specific nutrition knowledge areas (especially sports nutrition knowledge) or specific populations (e.g., adolescents, athletes) [e.g., (16–19)], only few validated instruments exist to measure nutrition knowledge in large population surveys.

Instruments that cover different types of nutrition knowledge are also rare (3). Some authors argue that a differentiation between declarative nutrition knowledge and procedural nutrition knowledge might be a promising approach for future research (2, 3, 20). Declarative knowledge is factual knowledge (“knowing that”) while procedural knowledge is knowledge of skills and strategies (“knowing how”) (2). Dickson-Spillmann et al. (2) developed and validated a short scale on nutrition knowledge (consumer nutrition knowledge scale—CoNKS) which encompasses both types of knowledge.

In Germany, the National Nutrition Survey (NVS) II (7) enquired single aspects of nutrition knowledge and confirmed that women, younger persons, and persons with a higher socio-economic status have higher nutrition knowledge and that persons with higher nutrition knowledge eat more fruit and less meat and alcoholic drinks (21, 22). However, to our knowledge, a psychometrically validated instrument has not been applied previously in a population-based survey in Germany. Additionally, most previous studies did not distinguish between different types of knowledge.

Therefore, the aims of the current study were to investigate the declarative and procedural nutrition knowledge in the German population, its determinants and its associations with food consumption based on the NEMONIT study (23) and using the CoNKS (2) as a validated and comparable measurement.

## Methods

### Study Design and Participants

This cross-sectional analysis is based on the last survey year data of the NEMONIT study (2014/15). NEMONIT was designed as a longitudinal study to assess changes in food consumption and nutrient intake in Germany. Besides the repeated measurement of food consumption, the annual surveys also included questions on specific nutritional topics (e.g., nutrition knowledge) allowing further cross-sectional analyses. A detailed description of NEMONIT has been published previously (23).

The NEMONIT sample (*n* ~2,000) was recruited from participants of the German National Nutrition Survey (NVS) II (7) who confirmed their interest in taking part in further surveys. Over the course of the survey years, the sample underwent minor changes due to attrition so that in the final NEMONIT round of 2014/2015, a total of 1,572 participants with an age range of 22–80 years were interviewed. In this analysis, participants of whom data of the computer-assisted telephone interview (CATI) and two 24-h recalls were available, were included (*n* = 1,508). Among those, three respondents with more than two missing items in the nutrition knowledge questions were excluded from the analysis resulting in a total sample of *n* = 1,505.

### Socio-Demographic Characteristics and Lifestyle Factors

Information on socio-demographic characteristics and lifestyle factors was obtained in CATIs. Socio-demographic characteristics included sex, age, school education (highest school-leaving qualification, recoded to years spent in school), and socio-economic status (SES). SES was an index based on three characteristics (participants' education, net household income and employment status of the principal earner of the household; possible range: 3–25 points) and categorized into low, medium, or high (21).

With regard to lifestyle factors, questions on specific diets (e.g., vegetarian diet, dieting), self-rated healthiness of the own diet, self-rated health status, and smoking status were included. Body mass index (BMI) was calculated based on self-reported body weight and height and categorized according to the cut-off points provided by the World Health Organization (WHO) (24). Physical activity was assessed by asking participants to state their mild, moderate, and vigorous physical activity in h/week. After bisecting hours of mild activity and doubling hours of vigorous activities, all activities were summed up as single measure for overall physical activity per week. This figure was compared to the WHO recommendation for physical activity (25) and categorized into inactive (0 h), active below recommendations (>0 and <2.5 hours) and active in agreement with the recommendations (≥2.5 h).

### Nutrition Knowledge

Nutrition knowledge was measured using an adapted version of the consumer nutrition knowledge scale (CoNKS) by Dickson-Spillmann et al. (2). This scale aims to measure nutrition knowledge in proximity to consumers by using well-known instead of scientific terms and includes both, declarative and procedural nutrition knowledge questions. In a Swiss sample it showed a good validity when compared to the General Nutrition Knowledge Questionnaire (GNKQ), the most widely used and validated questionnaire of nutrition knowledge, and in terms of subgroup differences (2). With only 20 questions (GNKQ: 89), the CoNKS can be regarded a short and efficient instrument to measure nutrition knowledge in larger population surveys. Therefore, it was considered suitable for the use in NEMONIT. Nevertheless, some adjustments of the scale were regarded necessary. These included the usage of food terms which are more common in Germany (e.g., “Emmental” instead of “Gruyère”) and the replacement of three items. Those items compared the healthiness of two alternatives (e.g., “Pasta with tomato sauce is healthier than pasta with mushroom and cream sauce”), which contradicts the view that it is the overall choice of foods and dishes, which makes a diet healthy or unhealthy rather than a food or dish *per se*. They were replaced by self-developed items comparing the nutrients of foods (Table 1). However, the item comparing the healthiness of brown and white sugar was kept, since in this case two variants of the same product are compared but consumers are expected to often misinterpret brown sugar as healthier due to the lower degree of product processing.

**Table 1:** Items of the adapted version of the consumer nutrition knowledge scale (CoNKS) and response behavior of the participants in the NEMONIT study 2014/15^a^, sorted by subscales and percentage of correct responses.

**Item**	**True/False**	**% of participants with**		
		**correct answer**	**wrong answer**	**don't know/missing**
**Procedural nutrition knowledge (0–7 points)**				
If you have eaten high-fat foods, you can reverse the effects by eating apples	F	91	4	5
A healthy meal should consist of half meat, a quarter vegetables and a quarter side dishes	F	78	21	1
Fat is always bad for your health; you should therefore avoid it as much as possible	F	71	28	1
A balanced diet implies eating all foods in the same amounts	F	69	29	1
For a healthy nutrition, dairy products should be consumed in the same amounts as fruit and vegetables	F	64	31	4
Brown sugar is much healthier than white sugar	F	60	31	9
To eat healthily, you should eat less fat. Whether you also eat more fruit and vegetables does not matter	F	55	42	2
**Declarative nutrition knowledge on nutrients (0–7 points)**				
Oily fish (salmon, mackerel) contain healthier fats than red meat	T	85	9	6
Lentils contain only few useful nutrients, therefore their health benefit is not great	F	81	8	10
^b^Meat sausage contains more salt than cream cheese	T	76	16	9
^b^Fruit muesli contains more fiber than cornflakes	T	73	17	10
Skimmed milk contains fewer minerals than full-fat milk	F	64	24	12
The health benefit of fruit and vegetables lies alone in the supply of vitamins and minerals	F	64	33	3
^b^Dairy products contain more saturated fats (fatty acids) than vegetable oils	T	37	44	19
**Declarative nutrition knowledge on calories (0–6 points)**				
If cream is whipped it contains less calories than in its liquid form	F	91	5	4
Bacon contains more calories than ham	T	80	15	4
Fat contains fewer calories than the same amount of fiber	F	79	11	11
The same amount of beef steak and chicken breast contains equally many calories	F	66	23	11
A sandwich with mozzarella contains as many calories as the same sandwich with Emmental/Swiss cheese	F	64	21	15
The same amount of sugar and fat contains equally many calories	F	60	25	15

a *NEMONIT study, survey year 2014/2015 (n = 1,505)*.

b *replaced items*.

The nutrition knowledge items were asked in randomized order during the CATI at the end of the second 24-h recall. For scale construction, items were recoded with correct answers taking the value “1” and incorrect answers, “don't know” answers and missing values taking the value “0.” Nutrition knowledge (CoNKS Total) was calculated as the sum of the 20 items, yielding a range of 0 to 20 points. Internal consistency of the scale was measured with Cronbach's Alpha (α = 0.59).

To analyse different types of knowledge, three subscales were formed based on content considerations: one scale for procedural nutrition knowledge (7 items, hence 0–7 points), one scale for declarative knowledge on nutrient contents (7 items, 0–7 points) and one scale for declarative knowledge on calorie content (6 items, 0-6 points) (Table 1). This approach differs from the validation study by Dickson-Spillmann et al. (2) where only one overall scale was used. The authors designed this scale to measure both, declarative and procedural knowledge, but did not distinguish the knowledge types in their analysis. Dickson-Spillmann et al. (2) did not theoretically distinguish between declarative knowledge on nutrient and calorie content either. However, we assumed that knowledge on nutrients and knowledge on calories could be different types of nutrition knowledge. Since the three subscales were based on content considerations, the results are exploratory in nature.

### Food Consumption and Diet Quality

Food consumption (g/d) was assessed with two 24-h recalls conducted on randomly drawn non-consecutive days (at least 1 week apart) by phone using the software EPIC-Soft (26) (renamed GloboDiet in 2014) as described in detail by Gose et al. (23). Energy and nutrient intakes were calculated based on the German Nutrient Database (BLS, version 3.02) (27).

Diet quality was evaluated using the Healthy Eating Index-NVS II (HEI-NVS II) adapted to 24-h recalls. The HEI-NVS II compares ten components of food consumption or nutrient intake [e.g., “fruit/fruit products,” “meat/meat products”„ “fat (% of energy intake)”] with food-based dietary guidelines of the German Nutrition Society (28) and national reference values for nutrient intake (29). It can take values from 0 to 110 points, whereby higher scores indicate a better agreement with the recommendations. Further information on the HEI-NVS II can be obtained from Gose et al. (23) and Wittig and Hoffmann (30).

### Data Analysis

Descriptive statistics are provided as means with standard deviations (SD) for metric variables and percentages for categorical variables. Since nutrition knowledge was not normally distributed, differences in nutrition knowledge between groups were tested using Mann–Whitney *U*-test or Kruskal–Wallis test as appropriate. Spearman's Rho correlations were calculated to examine the association of nutrition knowledge and its subscales with food consumption and HEI-NVS II. Multiple linear regressions were used to examine whether associations were independent from socio-demographic factors (sex, age in years, SES index).

Data analysis was performed using SAS 9.3 (SAS Institute, Inc.) and level of significance for all analyses was set at *P* < 0.05 (two-sided).

## Results

### Sample

Table 2 shows the socio-demographic characteristics and lifestyle factors of the study sample. The study sample includes a higher proportion of females than males. Participants had a mean age of 57 years and the majority achieved higher school education and were assigned to the medium SES class. The percentage of self-defined vegetarians and pesco-vegetarians was 3 and 7% of the participants were dieting. The majority rated their nutritional behavior as predominantly healthy while the HEI-NVS II indicates that there is need of improvement in the diet of a large majority of the participants (Table 2).

**Table 2:** Socio-demographic and lifestyle characteristics of NEMONIT study 2014/15^a^ participants.

	***n***	**% (Mean, SD)**
**Sex**		
Men	638	42.4
Women	867	57.6
**Age**		
Mean		(56.8)
SD		(14.2)
22–34 years	130	8.6
35–50 years	370	24.6
51–64 years	501	33.3
65–80 years	504	33.5
**School education**		
Up to 9 years	364	24.2
10 years	503	33.4
12 or 13 years	638	42.4
**SES**		
Mean		(15.0)
SD		(3.5)
Low	170	11.3
Medium	768	51.0
High	567	37.7
**Specific diet**		
Vegetarian (incl. pesco-vegetarian) diet	47	3.1
Dieting (e.g., to lose weight or due to an illness)	106	7.0
**Self-rated nutritional behavior**		
Very healthy	111	7.4
Predominantly healthy	1,237	82.2
Less healthy/not healthy	155	10.3
Missing	2	0.1
**HEI-NVS II**		
Mean		(67.9)
SD		(10.0)
Good (>88 points)^b^	26	1.7
In need of improvement (>55 and ≤88 points)^b^	1,326	88.1
Poor (≤55 points)^b^	153	10.2
**Body mass index**		
Mean		(26.0)
SD		(4.6)
Underweight	16	1.1
Normal weight	681	45.2
Preobese	587	39.0
Obese	221	14.7
**Self-rated health**		
Good	1,163	77.3
Moderate	301	20.0
Poor	39	2.6
Missing	2	0.1
**Physical activity**		
Inactive	441	29.3
Active, below recommendations	391	26.0
Active, in agreement with recommendations	654	43.5
Missing	19	1.3
**Smoking**		
Smoker	165	11.0
Occasional smoker	42	2.8
Ex-smoker	521	34.6
Non-smoker	777	51.6

a *NEMONIT study, survey year 2014/2015 (n = 1,505); SES, socio-economic status (index combining participants' education, net household income, and employment status of the principal earner of the household); HEI-NVS II, Healthy Eating Index of the German National Nutrition Survey II*;

b *55 points ≜ 50% of total points, 88 points ≜ 80% of total points*.

### Nutrition Knowledge

On average, items of the nutrition knowledge scale were answered correctly by 70% of the participants. All except one item were answered correctly by more than half of the participants, indicating relatively easy items (in terms of scale construction). Participants' mean was 14.1 points for the CoNKS Total (SD 3.0, IQR 4), 4.9 points for the subscale procedural knowledge (SD 1.6, IQR 2), 4.8 points for knowledge on nutrients (SD 1.4, IQR 1), and 4.4 points for knowledge on calories (SD 1.2, IQR 2) (Table 3). The distribution of scores was skewed to the left in all scales (data not shown).

**Table 3:** Nutrition knowledge (CoNKS Total and subscales) by socio-demographic group^a^ in participants of the NEMONIT study 2014/15^b^.

	**CoNKS total**^****c****^		**Procedural knowledge**^****d****^		**Knowledge on nutrients**^****d****^		**Knowledge on calories**^****e****^	
	**Mean**	***P***	**Mean**	***P***	**Mean**	***P***	**Mean**	***P***
Total sample	14.1		4.9		4.8		4.4	
Sex		<0.001		<0.001		0.006		0.689
Males	13.6		4.6		4.7		4.4	
Females	14.4		5.1		4.9		4.4	
Age groups		<0.001		<0.001		<0.001		<0.001
22–34 years	15.3		5.6		5.0		4.7	
35–50 years	14.7		5.2		5.0		4.5	
51–64 years	14.4		5.0		4.9		4.5	
65–80 years	13.0		4.3		4.5		4.2	
School education		<0.001		<0.001		<0.001		<0.001
Up to 9 years	12.5		4.1		4.2		4.1	
10 years	14.2		4.9		4.8		4.4	
12 or 13 years	15.0		5.3		5.1		4.5	
SES class		<0.001		<0.001		<0.001		<0.001
Low	12.2		4.1		4.1		4.1	
Medium	13.9		4.8		4.7		4.4	
High	14.9		5.3		5.1		4.5	

a *Mann–Whitney U-test (variables with two levels) or Kruskal–Wallis test (variables with more than two levels)*.

b *NEMONIT study, survey year 2014/2015 (n = 1,505); SES, socio-economic status; CoNKS, consumer nutrition knowledge scale*.

c *CoNKS Total scale ranges from 0 (no question answered correctly) to 20 (all questions answered correctly)*.

d *CoNKS subscale procedural knowledge and knowledge on nutrients each range from 0 (no question in the respective subsection answered correctly) to 7 (all questions in the respective subsection answered correctly)*.

e *CoNKS subscale knowledge on calories ranges from 0 (no question in this subsection answered correctly) to 6 (all questions in this subsection answered correctly)*.

Some areas for knowledge enhancement can be identified when looking at the single items (Table 1). First, the health benefits of fruit and vegetable consumption do not seem to be sufficiently well-known to the general public. Forty two percentage of the participants assumed the following statement to be correct: “To eat healthily, you should eat less fat. Whether you also eat more fruit and vegetables does not matter.” Additionally, about one third of the participants thought the statement “The health benefit of fruit and vegetables lies alone in the supply of vitamins and minerals” to be true. Second, deficits in knowledge concerning the meaning of a balanced diet became apparent. About one third of the participants thought the following statements to be true: “A balanced diet implies eating all foods in the same amounts” and “For a healthy nutrition, dairy products should be consumed in the same amounts as fruit and vegetables.” Furthermore, about one fifth of the participants thought that “A healthy meal should consist of half meat, a quarter vegetables and a quarter side dishes.” Notably, the questions on a balanced diet and on the health benefits of fruit and vegetables received only few “don't know” answers, compared to questions on specific nutrients and calories. This means that participants were confident about their knowledge in this area, although they more often gave wrong answers than in other areas. Third, the question on saturated fatty acids in dairy products vs. vegetable oils revealed large uncertainties in this area (37% correct answers).

### Socio-demographic Characteristics and Nutrition Knowledge

Nutrition knowledge differed significantly between socio-demographic groups (Table 3). Women had a higher nutrition knowledge than men, except for knowledge on calories. Nutrition knowledge was higher in younger age groups and in groups with higher school education and higher SES. The results were confirmed in multiple linear regressions (data not shown).

### Lifestyle Factors and Nutrition Knowledge

Nutrition knowledge was higher among individuals following a vegetarian diet, having a normal weight and being physically active (Table 4). This also applied to the subscales of procedural knowledge and knowledge on nutrients but not to the scale measuring knowledge on calories (except for sports activities).

**Table 4:** Nutrition knowledge (CoNKS Total and subscales) by nutrition and health behaviour^a^ in participants of the NEMONIT study 2014/15^b^.

	**CoNKS Total**		**Procedural knowledge**		**Knowledge on nutrients**		**Knowledge on calories**	
	**Mean**	***P***	**Mean**	***P***	**Mean**	***P***	**Mean**	***P***
Vegetarian (incl. pesco-vegetarian) diet		<0.001^†^		<0.001^†^		<0.001^†^		0.604
Yes	16.1		6.1		5.6		4.5	
No	14.0		4.9		4.8		4.4	
Dieting (e.g., to lose weight or due to an illness)		0.016		0.036		0.131		0.083
Yes	13.3		4.5		4.6		4.2	
No	14.1		4.9		4.8		4.4	
Healthiness of diet		0.182		0.105		0.173		0.923
Very healthy/ predominantly healthy	14.1		4.9		4.8		4.4	
Less healthy/ not healthy	13.8		4.7		4.7		4.4	
Subjective health status		0.096		0.044		0.200		0.646
Very good/ good	14.2		4.9		4.8		4.4	
Moderate/ poor/very poor	13.8		4.7		4.7		4.4	
Body mass index		<0.001^†^		<0.001^†^		<0.001^†^		0.655
Normal weight	14.6		5.2		5.0		4.4	
Underweight, overweight	13.7		4.7		4.7		4.4	
Sport activities		<0.001^†^		<0.001^†^		<0.001		0.022
Active	14.3		5.0		4.9		4.4	
Inactive	13.5		4.6		4.6		4.3	
Smoking status		0.079		0.052		0.059		0.355
Non-smoker/ex-smoker	14.1		4.9		4.8		4.4	
Smoker/occasional smoker	13.7		4.7		4.6		4.4	

a *Mann–Whitney U-test*.

b *NEMONIT study, survey year 2014/2015 (n = 1,505); CoNKS, consumer nutrition knowledge scale*.

† *Significant difference confirmed in multiple linear regression analyses controlling for sex, age in years, and socio-economic status*.

The significant results of the bivariate analysis were generally confirmed in multiple linear regressions controlling for sex, age, and SES (data not shown).

### Nutrition Knowledge and Food Consumption

Nutrition knowledge was positively associated with the consumption of cereals/cereal products, vegetables, fruit/fruit products, and dairy products and negatively with the consumption of potatoes/potato products and meat/meat products (Table 5). However, correlations were rather weak with values between −0.14 and 0.12. Very similar results were observed when analyzing procedural knowledge and knowledge on nutrients separately. Knowledge on calories, however, was not associated with the consumption of cereals/cereal products, vegetables, fruit/fruit products, or meat/meat products.

**Table 5:** Association between nutrition knowledge (CoNKS Total and subscales) and food consumption (g/d)^a^ in participants of the NEMONIT study 2014/15^b^.

	**CoNKS Total**		**Procedural knowledge**		**Knowledge on nutrients**		**Knowledge on calories**	
	***r_***s***_***	***P***	***r_***s***_***	***P***	***r_***s***_***	***P***	***r_***s***_***	***P***
Bread	0.00	0.958	−0.03	0.227	0.03	0.227	−0.00	0.984
Cereals and cereal products	0.11	<0.001	0.14	<0.001	0.07	0.006	0.02	0.394
Potatoes and potato products	−0.06	0.029	−0.05	0.046	−0.00	0.860	−0.07	0.004^†^
Vegetables^c^	0.09	<0.001^†^	0.11	<0.001^†^	0.11	<0.001^†^	−0.04	0.173
Fruit and fruit products	0.10	<0.001^†^	0.10	<0.001^†^	0.09	<0.001^†^	0.01	0.630
Milk, dairy products, and cheese	0.12	<0.001^†^	0.07	0.009	0.13	<0.001^†^	0.06	0.023
Eggs	0.01	0.590	−0.01	0.635	0.03	0.300	0.02	0.514
Meat, meat products	−0.14	<0.001^†^	−0.14	<0.001^†^	−0.11	<0.001^†^	−0.05	0.070
Fish, fish products, and seafood	0.03	0.194	0.03	0.259	0.05	0.077	−0.01	0.588

a *Spearman's Rho correlations*.

b *NEMONIT study, survey year 2014/2015 (n = 1,505); CoNKS, consumer nutrition knowledge scale*.

c *Including vegetable products, mushrooms, and pulses*.

† *Significant association confirmed in multiple linear regression analyses controlling for sex, age in years, and socio-economic status index*.

In multiple linear regressions, the associations between nutrition knowledge and its subscales with consumption of vegetables, fruit/fruit products, dairy products, and meat/meat products were largely confirmed.

### Nutrition Knowledge and Diet Quality

With increasing values on nutrition knowledge, respondents also had increasing values on the HEI-NVS II (Spearman's Rho correlation coefficient: *r*_*s*_ = 0.16, *p* < 0.001). This also applied to the subscales procedural knowledge (*r*_*s*_ = 0.15, *p* < 0.001) and knowledge on nutrients (*r*_*s*_ = 0.16, *p* < 0.001), but not to knowledge on calories (*r*_*s*_ = 0.02, *p* = 0.440). However, correlations were rather weak and in a simple linear regression model, nutrition knowledge explained only 3% (procedural knowledge: 2%, knowledge on nutrients: 3%) of the variance in HEI-NVS II (data not shown).

Similar to nutrition knowledge, HEI-NVS II was higher among women and among groups with higher school education and higher SES. However, HEI-NVS II increased with age while nutrition knowledge decreased with age. Therefore, multiple linear regressions were performed again to examine whether the association between nutrition knowledge and HEI-NVS II was independent of sex, age, and SES (Table 6). The results confirmed an independent association between nutrition knowledge and HEI-NVS II. However, they also showed that SES was no longer a significant predictor of HEI-NVS II when nutrition knowledge was entered into the model. Therefore, nutrition knowledge may partly mediate the effect of SES on healthy eating.

**Table 6:** Associations of socio-demographic characteristics and nutrition knowledge (CoNKS Total and selected subscales) with HEI-NVS II^a^ in participants of the NEMONIT study 2014/15^b^.

	**Unstandardized**	***P***
	**regression**	
	**coefficient**	
**Model: socio-demographic characteristics only (Adj**. ***R**^2^* **=** **0.024)**		
Female sex	2.986	<0.001
Age (in years)	0.055	0.003
SES index	0.177	0.019
**Model: socio-demographic characteristics and nutrition knowledge (CoNKS Total) (Adj**. ***R**^2^* **=** **0.051)**		
Female sex	2.391	<0.001
Age (in years)	0.080	<0.001
SES index	0.023	0.760
CoNKS Total	0.620	<0.001
**Model: socio-demographic characteristics and CoNKS subscale procedural knowledge (Adj**. ***R**^2^* **=** **0.043)**		
Female sex	2.407	<0.001
Age (in years)	0.077	<0.001
SES index	0.065	0.400
Procedural knowledge	0.924	<0.001
**Model: socio-demographic characteristics and CoNKS subscale knowledge on nutrients (Adj**. ***R**^2^* **=** **0.051)**		
Female sex	2.630	<0.001
Age (in years)	0.064	<0.001
SES index	0.052	0.500
Knowledge on nutrients	1.289	<0.001

a *Multiple linear regression analysis with HEI-NVS II as dependent and socio-demographic characteristics and nutrition knowledge (CoNKS Total), respectively, CoNKS subscales procedural knowledge and knowledge on nutrients as independent variables*.

b *NEMONIT study, survey year 2014/2015 (n = 1,505); SES, socio-economic status; CoNKS, consumer nutrition knowledge scale; HEI-NVS II, Healthy Eating Index of the German National Nutrition Survey II*.

## Discussion

This analysis of nutrition knowledge in adults based on NEMONIT and using an adapted version of the CoNKS showed several key results:

1. Areas for knowledge enhancement could be observed in the assessment of the health benefits of fruit and vegetable consumption, in the understanding of the concept of a balanced diet as well as regarding the knowledge on saturated fatty acids.
2. Nutrition knowledge was higher among individuals who were female, younger, had higher SES or showed a more health conscious lifestyle.
3. Nutrition knowledge was positively associated with a favorable food consumption.
4. Analyses of subscales of nutrition knowledge yielded similar results for procedural nutrition knowledge and knowledge on nutrients but not for knowledge on calories.

### Areas for Knowledge Enhancement

In accordance with the results from Dickson-Spillmann et al. (2), the present results indicate that the health benefits of fruit and vegetable consumption do not seem to be sufficiently well-known to the public. This is unexpected since in Germany, as in many other countries, large efforts have been made to promote the consumption of fruit and vegetables, which is below the official recommendations. Hence, improved strategies are needed to communicate the (numerous) advantages of fruit and vegetables and to increase knowledge as basis for intention and action to promote their consumption.

Also in agreement with Dickson-Spillmann et al. (2), the present results showed that knowledge concerning the composition of a healthy and balanced diet could be improved. Much educational work has been done in this area, too. Among others, food circles or pyramids are a common way to present the principles of a balanced diet and are usually well disseminated and known to the public (31, 32). However, consumers seem to have difficulties to keep in mind, interpret, and apply these principles (33, 34). Nutrition education in this area might benefit from providing more common and practically relevant examples.

Additionally, knowledge on saturated fatty acids seems relatively low. This result reinforces international findings ascertaining knowledge deficits with regard to types of dietary fats (35, 36). Types of fat, their health implications and their sources could be another focus of nutrition education initiatives.

Although this research identified some important areas to address in nutrition education, it simultaneously indicates that an increase in nutrition knowledge alone will not substantially improve dietary behavior (see below).

### Associations of Socio-demographic and Lifestyle Factors With Nutrition Knowledge

That nutrition knowledge is higher in women, normal weight and physically active persons as well as among those with higher socio-economic status (or its indicators such as education or employment status) has already been observed in a number of earlier studies and was discussed previously (8, 10, 36–39).

The relationship between age and nutrition knowledge, however, was contradictory across studies (2, 8–10). Here, similar to Dickson-Spillmann et al. (2), age was negatively associated with nutrition knowledge. The CoNKS assessed knowledge based on insights and recommendations of the last years, e.g., to correctly reject the item “Fat is always bad for your health; you should therefore avoid it as much as possible” one must recognize that nowadays a distinction according to the type of fat is made. Older respondents might have more difficulties to obtain the necessary information and to integrate these into their already well-established concept of a healthy nutrition. Given the contradictory results on the association between age and nutrition knowledge, more research might be needed to examine if people in different age groups or stages of live have access to, understand and are able to practically implement the knowledge necessary to choose a healthy diet.

In this study, self-defined vegetarians (including pesco-vegetarians) compared to non-vegetarians had a higher nutrition knowledge. Up to now, there are only few and inconsistent studies on differences in nutrition knowledge among vegetarians and non-vegetarians (40). But Hoffman (40) argues that vegetarians often become “nutrition educators” since they are regularly confronted with nutrition-related questions, like where to get protein or iron in a vegetarian diet. However, the results of the present study should be interpreted with caution due to the low proportion of vegetarians.

### Associations of Nutrition Knowledge With Food Consumption and Diet Quality

Participants with higher nutrition knowledge ate more favorable (e.g., vegetables, fruit/fruit products) and less unfavorable foods (e.g., meat/meat products) and showed a higher diet quality overall. Although significant associations in the expected direction were observed, the correlations between nutrition knowledge and food consumption were low in this study, also when compared to the validation study of the CoNKS (2). The results support those findings observing only a weak relationship between nutrition knowledge and dietary behavior (3). This indicates that an increase in nutrition knowledge alone seems unlikely to provoke large improvements in dietary behavior. Dietary behavior is complex and influenced by a number of different factors (41).

### Types of Nutrition Knowledge

The separate analysis of procedural knowledge and knowledge on nutrients provided similar results as the analysis of nutrition knowledge in total. Knowledge on calories, however, seems to be a different kind of knowledge. Research on different types of knowledge is rare, but Grunert et al. (42) also observed that knowledge on the calorie content of foods was unrelated to knowledge on dietary recommendations and sources of nutrients. For future studies it might be helpful to examine the construct of nutrition knowledge and its dimensions more closely to get a better understanding of which types of knowledge might be relevant for a healthy dietary behavior in the population.

According to our results, knowledge on calories does not seem helpful in making healthy food choices. Contrary to what we would theoretically expect, it was not associated with BMI either. Knowledge on the caloric content of macronutrients, foods and meals might be too technical to be translated into a diet with adequate energy intake. Our results suggest that it might be advisable to include more information on, for e.g., the contribution of a meal to a balanced diet, in the commonly used media rather than just information on calories.

### Strengths and Limitations

This study has several strengths. First, it explored nutrition knowledge based on a large sample of the German adult population. This allowed using multivariate analyses to examine group differences and associations independent from socio-demographic factors. Second, the study used a scale which showed a good ability to distinguish between nutrition-literate and lay respondents. Although some essential modifications were made, low associations between nutrition knowledge and dietary behavior are unlikely to result from incapacity of the scale to distinguish between participants with different grades of nutrition knowledge. The measurement also allowed investigating both declarative and procedural nutrition knowledge. Low associations therefore cannot be attributed to a mere assessment of declarative knowledge, which was assumed by some authors to have a lower relevance for dietary behavior (2, 3). Third, food consumption was assessed with two 24-h recalls, which is in accordance with the requirements of the European Food Safety Authority regarding collection of national food consumption data (43). Fourth, the healthy eating index was calculated from actual food consumption as an outcome variable to represent compliance with dietary guidelines. This was supposed to be an outcome more closely related to nutrition knowledge, but still associations prove to be low.

Some limitations of the study also need to be considered. First, the study sample was biased toward women, older persons and persons with a higher SES (23) and respondents who took part in NEMONIT for several years might be especially interested in nutrition topics. Based on this sample, nutrition knowledge might be overestimated and the discussed deficits in nutrition knowledge might be higher in the general population.

Second, the measurement of nutrition knowledge includes some uncertainties. Although nutrition knowledge was measured using a previously validated instrument, Cronbach's Alpha, which is used to assess the internal consistency of a scale, was low (α = 0.59). The value could not be substantially increased by deleting an item and the correlations between some items were very low. This could indicate that nutrition knowledge is a heterogeneous construct with different dimensions. Another possible explanation for a low Cronbach's Alpha would be a very homogenous sample. As previously mentioned, NEMONIT respondents might consistently have a higher interest in nutrition topics. Nutrition knowledge in the sample was high with a relatively low standard deviation. This might restrict the ability of the study to find large associations between nutrition knowledge and dietary behavior.

Finally, it is important to note that no causal relationships can be implied from the cross-sectional analysis.

## Conclusion

The present study identified areas for knowledge enhancement in the assessment of the health benefits of fruit and vegetable consumption, in the understanding of the concept of a balanced diet as well as regarding the knowledge on saturated fatty acids. These topics might be most relevant for future nutrition education efforts. However, this study also supports a number of previous studies observing significant but weak associations between nutrition knowledge and dietary behavior. This indicates that an increase in nutrition knowledge through nutrition education alone is unlikely to provoke large improvements in dietary behavior. From health and sustainability literature, it is well-known that knowledge is usually not directly translated into action. Instead, behavior is complex and influenced by a number of different factors. Research should find ways to address the complexity of dietary behavior and to identify the most important factors that need to be addressed to improve dietary behavior of the population.

## Data Availability Statement

The datasets presented in this article are not readily available because according to the regulations for the use of NVS II- and NEMONIT-study data, the datasets of the NEMONIT study are only available for universities, public and/or publicly funded scientific research institutions. Furthermore, a general essential prerequisite is the pure scientific use of the data, excluding any commercial use of the data and of the derived results. Requests to access the datasets should be directed to the corresponding author, ingrid.hoffmann@mri.bund.de.

## Ethics Statement

Ethical review and approval was not required for the study on human participants in accordance with the local legislation and institutional requirements. The patients/participants provided their written informed consent to participate in this study.

## Author Contributions

FK analyzed and interpreted the data and drafted the manuscript. IH was involved in data interpretation and manuscript preparation. EC initiated and conceptualized the research, was involved in data interpretation and manuscript preparation and was responsible for the final content. All authors read and approved the final version of the manuscript.

## Conflict of Interest

The authors declare that the research was conducted in the absence of any commercial or financial relationships that could be construed as a potential conflict of interest.
